# Bacterial Genome-Wide Association Identifies Novel Factors That Contribute to Ethionamide and Prothionamide Susceptibility in Mycobacterium tuberculosis

**DOI:** 10.1128/mBio.00616-19

**Published:** 2019-04-23

**Authors:** Nathan D. Hicks, Allison F. Carey, Jian Yang, Yanlin Zhao, Sarah M. Fortune

**Affiliations:** a Department of Immunology and Infectious Diseases, Harvard T. H. Chan School of Public Health, Boston, Massachusetts, USA; b Department of Pathology, Massachusetts General Hospital, Boston, Massachusetts, USA; c MOH Key Laboratory of Systems Biology of Pathogens, Institute of Pathogen Biology, and Centre for Tuberculosis, Chinese Academy of Medical Sciences and Peking Union Medical College, Beijing, China; d National Center for Tuberculosis Control and Prevention, Chinese Center for Disease Control and Prevention, Beijing, China; e Ragon Institute of MGH, MIT, and Harvard, Cambridge, Massachusetts, USA; f Broad Institute of MIT and Harvard, Cambridge, Massachusetts, USA; Washington University in St. Louis School of Medicine

**Keywords:** genome-wide association study, *Mycobacterium tuberculosis*, antibiotic resistance, ethionamide, genome analysis, prothionamide

## Abstract

In Mycobacterium tuberculosis, recent genome-wide association studies have identified a novel constellation of mutations that are correlated with high-level drug resistances. Interpreting the functional importance of the new resistance-associated mutations has been complicated, however, by a lack of experimental validation and a poor understanding of the epistatic factors influencing these correlations, including strain background and programmatic variation in treatment regimens. Here we perform a genome-wide association analysis in a panel of Mycobacterium tuberculosis strains from China to identify variants correlated with resistance to the second-line prodrug ethionamide (ETH). Mutations in a bacterial monooxygenase, Rv0565c, are significantly associated with ETH resistance. We demonstrate that Rv0565c is a novel activator of ETH, independent of the two known activators, EthA and MymA. Clinically prevalent mutations abrogate Rv0565c function, and deletion of Rv0565c confers a consistent fitness benefit on *M. tuberculosis* in the presence of partially inhibitory doses of ETH. Interestingly, Rv0565c activity affects susceptibility to prothionamide (PTH), the ETH analog used in China, to a greater degree. Further, clinical isolates vary in their susceptibility to both ETH and PTH, to an extent that correlates with the total expression of ETH/PTH activators (EthA, MymA, and Rv0565c). These results suggest that clinical strains considered susceptible to ETH/PTH are not equally fit during treatment due to both Rv0565c mutations and more global variation in the expression of the prodrug activators.

## Abstract (precis)

Phenotypic antibiotic susceptibility testing in Mycobacterium tuberculosis is slow and cumbersome. Rapid molecular diagnostics promise to help guide therapy, but such assays rely on complete knowledge of the molecular determinants of altered antibiotic susceptibility. Recent genomic studies of antibiotic-resistant *M. tuberculosis* have identified several candidate loci beyond those already known to contribute to antibiotic resistance; however, efforts to provide experimental validation have lagged. Our study identifies a gene (Rv0565c) that is associated with resistance to the second-line antibiotic ethionamide at a population level. We then use bacterial genetics to show that the variants found in clinical strains of *M. tuberculosis* improve bacterial survival after ethionamide exposure.

## INTRODUCTION

Even as the global incidence and mortality of tuberculosis disease (TB) have steadily decreased over the last several years, the incidence of multidrug-resistant TB (MDR-TB) is projected to rise over the next decade (1). The discrepancy in these trends is in part due to large differences in the efficacy of antibiotic treatment for drug-susceptible tuberculosis (DS-TB) versus MDR-TB; DS-TB is treated for 6 months with a combination of four potent first-line antibiotics capable of achieving a durable cure in ∼95% of patients (2, 3), while MDR-TB is treated using more complex and longer regimens that have significantly lower success rates (4).

Many factors are likely to contribute to the outcome of MDR-TB treatment, including the ability to choose antimicrobials to which the bacterium is fully susceptible. Regimen decisions are complicated by limited capacity to phenotypically measure second-line drug resistances in many parts of the world and an incomplete understanding of the genetic basis of altered treatment responses that would allow genotypic susceptibility testing (5). Defining a robust genotype-to-phenotype map for drug susceptibility in Mycobacterium tuberculosis has become feasible as whole-genome sequencing is applied to strains selected for antibiotic resistance *in vitro* as well as large numbers of clinical isolates for which drug susceptibility patterns are known (6, 7). Recently, genome-wide association (GWA) approaches have been applied to identify genetic determinants of drug resistance in several bacterial pathogens (8). These studies benefit from directly examining mutations that have evolved in the host environment with physiological levels and timing of antibiotic exposure. However, because most novel associations identified in these studies to date have not been experimentally validated, there is limited understanding of what phenotypes these mutations confer and how to incorporate these associations into treatment decisions. Determining the relationship between genotype and phenotype has been complicated by the inconsistent findings between GWA studies, perhaps due to differences in the methods used or biological differences between strain sets. Furthermore, recent work has suggested that many different forms of altered susceptibility are possible which are not captured by standard phenotypic drug susceptibility testing (9, 10).

Using various genome-wide association approaches, three recent studies have specifically looked for genetic loci with mutations correlating with resistance to the antimycobacterial agent ethionamide (ETH) (11–13). ETH, or its analog prothionamide (PTH), is an orally available second-line agent often incorporated into regimens for multidrug-resistant TB (14). ETH is a prodrug activated by the bacterial monooxygenase EthA (*Rv3854c*) (15) which then forms an ETH-NAD adduct capable of inhibiting the production of essential mycolic acids by targeting the enoyl acyl carrier protein reductase InhA (16). A wide range of mutations in *ethA*, in the promoter of the *fabG1-inhA* operon causing overexpression of *inhA*, or within *inhA* itself alter susceptibility to ETH (17, 18); however, these mutations do not explain resistance in all clinical isolates (5), suggesting that additional genes contribute to ETH susceptibility. Moreover, not all strains with EthA loss-of-function mutations are phenotypically ETH resistant (19), further suggesting that other ETH activators may exist. Recurrent loss-of-function mutants in genes encoding two additional bacterial monooxygenases, *mymA* (*Rv3083*) and *Rv0565c*, were identified in a large clinical data set from Russia (19), and a recent study implicated *mymA* in ETH susceptibility during *in vitro* growth, where mutation of either *ethA* or *mymA* conferred intermediate resistance to ETH, whereas the double mutant conferred a much higher level of resistance (20). In this study, *Rv0565c* deletion did not similarly alter ETH susceptibility (20). However, another study suggested that *Rv0565c* mutations were specifically correlated with *ethA* mutations in only one bacterial lineage (13) and proposed that *Rv0565c* mutations may be compensatory mutations reflecting the complexity of assessing genetic contributions to drug susceptibility.

Here we sought to identify genes in which mutations are significantly correlated with ETH resistance and determine their impact on ETH susceptibility. We used an algorithm that we recently developed, phyOverlap (9), to assess a set of 145 strains from China that had been phenotyped for ETH resistance. The most significant novel association with ETH resistance was *Rv0565c*. Given the strength of the association and the mixed data in the literature, we here experimentally define the role of Rv0565c in ETH/PTH susceptibility in M. tuberculosis. We demonstrate that Rv0565c mutations provide a subtle but consistent survival benefit in the face of ETH treatment in both Euro-American and East Asian lineage strains and have an unexpectedly stronger effect in PTH, which is used in place of ETH in China. We further demonstrate that clinical strains that are considered ETH susceptible vary in their total expression of the three ETH activators, EthA, MymA, and Rv0565c, and that total activator expression correlates well with bacterial growth restriction in sub-MICs of drug.

## RESULTS

### Mutations in Rv0565c are associated with ethionamide resistance in clinical isolates.

**FIG 1:**
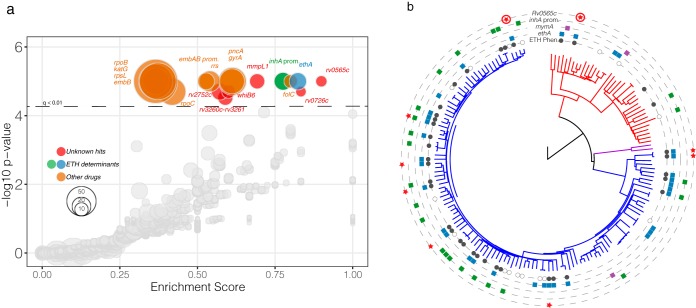
Genome-wide association with ETH resistance identifies *Rv0565c*. (a) Genome-wide associations for all genes and intergenic regions containing variants among the 145 strains with ETH susceptibility testing. Each gene or intergenic region is plotted by the enrichment score (see Materials and Methods) and phyOverlap *P* value. (b) The genome-wide SNP phylogeny of all 161 strains from Zhang et al. (11) rooted with *M. canettii*. Phenotypic ETH resistance is indicated in the innermost circle where open circles are strains for which phenotyping was not performed. Variants in the monooxygenase genes *ethA*, *mymA*, and *Rv0565c* as well as the promoter of *inhA*, excluding common-lineage-defining SNPs, are indicated in each ring. Circled stars in the *Rv0565c* ring represent isolates with indel mutations not included in the SNP analysis.

To identify genes and intergenic regions in which mutations are associated with ETH resistance in clinical isolates, we used phyOverlap (9) to perform genome-wide association testing on a set of sequenced *M. tuberculosis* strains from China for which drug susceptibility phenotypes were available (11). Eighteen genes and intergenic regions were associated with ETH resistance at the genome-wide level (*q* < 0.01). Among these were several genes in which mutations are known to confer resistance to ETH, including *ethA* and the *inhA* promoter (Fig. 1a). Genes conferring resistance to other clinically utilized antibiotics, including rifampin and isoniazid, were also identified, which is expected given that *M. tuberculosis* genomes are in complete linkage and ETH is used only in the setting of preexisting drug resistances.

Among the significant genetic associations, mutations in *Rv0565c* were most strongly enriched in ETH-resistant isolates, almost perfectly overlapping with phenotypic ETH resistance. Mutations in this gene were identified as enriched in strains with ETH, ofloxacin, kanamycin, and capreomycin resistances in the original analysis of these strains (11). Among the 36 ETH-resistant strains in this data set, there were 6 independent SNPs in *Rv0565c*. No *Rv0565c* mutations were found in susceptible strains except the R110H variant that is shared by all isolates from the phylogenetically defined lineage 2, also known as the East Asian lineage (21). One additional significant gene, *Rv0726c*, mostly coincided with ETH resistance, while the remaining genes, including *mmpL1*, *whiB6*, and *Rv2752c*, and the *Rv3260c-Rv3261* intergenic region were grouped closer to known determinants of resistance to other antibiotics (Fig. 1a), suggesting they may play a role in resistance to another agent.

To further characterize the genomic context in which *Rv0565c* mutations are found, we manually examined the sequence of *Rv0565c, ethA*, *mymA*, and the *inhA* promoter in all 145 strains for which ETH susceptibility was tested as well as the additional 16 strains in which no ETH phenotype was available (Fig. 1b; see also Table S1a to c in the supplemental material). We identified an additional two ETH-resistant isolates with frameshift mutations in *Rv0565c*, which were not included in the genome-wide SNP analysis (Fig. 1b, circled stars). Although *Rv0565c* mutations (except R110H) occurred specifically within ETH-resistant isolates, in every case these mutations cooccurred with *ethA* mutations (Fig. S1). Overall, *mymA* mutants were rare in this data set, occurring in only 3 isolates, two of which were scored as ETH susceptible while the other one was not tested. To examine whether *Rv0565c* mutations always occur in the background of *ethA* mutations, we expanded the analysis of *Rv0565c* and *ethA* to an additional 549 strains from China for which ETH susceptibility was not determined (9). Among these strains, we identified 20 strains with *Rv0565c* mutations, excluding the R110H site, of which only 10 strains also contained mutations within *ethA*, suggesting that *Rv0565c* mutations do not always occur subsequent to *ethA* mutation (Fig. S1; Table S2).

10.1128/mBio.00616-19.1 FIG S1 The overlap of *Rv0565c* and *ethA* mutations among strains from Zhang et al. (11) and Hicks et al. (9). The common, lineage-associated mutations *Rv0565c-R110H*, *ethA-S266R*, and *ethA-P334A* were excluded from this analysis. Download FIG S1, PDF file, 0.8 MB. Copyright © 2019 Hicks et al. 2019 Hicks et al. This content is distributed under the terms of the Creative Commons Attribution 4.0 International license.

10.1128/mBio.00616-19.7 TABLE S1 Complete list of *Rv0565c* (a), *mymA* (b), and *ethA* (c) mutations found in the 161 strains from Zhang et al. (11) included in Fig. 1b. Download Table S1, XLSX file, 0.01 MB. Copyright © 2019 Hicks et al. 2019 Hicks et al. This content is distributed under the terms of the Creative Commons Attribution 4.0 International license.

10.1128/mBio.00616-19.8 TABLE S2 Genotypes of *Rv0565c* and *ethA* variants in the 549 strains analyzed from Hicks et al. (9) (Fig. S1, right panel). Download Table S2, XLSX file, 0.01 MB. Copyright © 2019 Hicks et al. 2019 Hicks et al. This content is distributed under the terms of the Creative Commons Attribution 4.0 International license.

### Rv0565c expression confers hypersensitivity to ETH in Mycobacterium smegmatis by targeting *inhA*.

Based on sequence homology, Rv0565c is a Baeyer-Villiger monooxygenase in the same family as the known activators *ethA* and *mymA* (20). We hypothesized that Rv0565c may likewise be capable of activating ETH. To test this hypothesis, we first attempted to purify recombinant Rv0565c protein to assess biochemical function, but we were unable to obtain soluble protein. Therefore, we performed a genetic analysis of Rv0565c-related ETH susceptibility in the environmental mycobacterium M. smegmatis.

**FIG 2:**
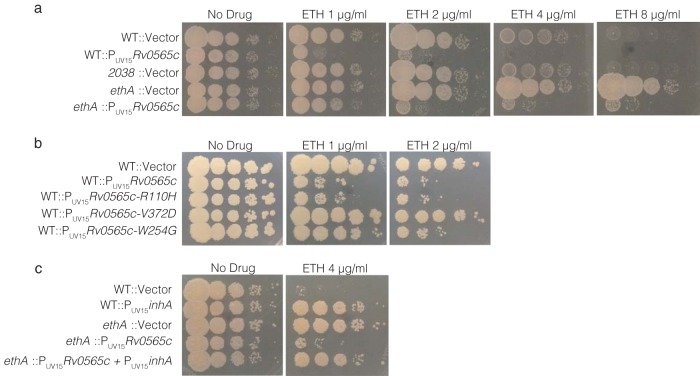
*Rv0565c* activates ETH to target *inhA* in M. smegmatis. (a to c) Growth of indicated M. smegmatis strains was monitored by spot plating of serial 10-fold dilutions of the indicated strains on solid medium containing various concentrations of ETH. Images were taken after 3 days of growth.

M. smegmatis contains genes homologous to *ethA* (*Msmeg_6440*) and *Rv0565c* (*Msmeg_2038*) but no *mymA* homolog. As expected, deletion of *ethA^Msmeg^* dramatically increased bacterial growth on increasing concentrations of ETH. Deletion of *Msmeg_2038* did not alter ETH susceptibility (Fig. 2a). However, constitutive expression of Rv0565c under the strong UV15 promoter hypersensitized M. smegmatis to ETH, even in the absence of *ethA^Msmeg^*, suggesting that Rv0565c can act as an independent activator of ETH (Fig. 2a). Consistent with the model that Rv0565c directly acts on ETH rather than altering mycolic acid metabolism, constitutive expression of Rv0565c failed to sensitize M. smegmatis to isoniazid (INH), which shares the same target as ETH but is activated by the bacterial catalase-peroxidase *katG* (Fig. S2).

10.1128/mBio.00616-19.2 FIG S2 Growth of indicated M. smegmatis strains was monitored by spot plating of serial 10-fold dilutions of the indicated strains on solid medium containing various concentrations of INH and ETH. Images were taken after 2.5 days of growth. Download FIG S2, PDF file, 8.4 MB. Copyright © 2019 Hicks et al. 2019 Hicks et al. This content is distributed under the terms of the Creative Commons Attribution 4.0 International license.

Although we identified two clinical isolates in which *Rv0565c* was the target of frameshifting mutations, several isolates contain SNPs for which the functional consequence is not obvious. Further, lineage 2 (East Asian) isolates all share an R110H variant in *Rv0565c* which could alter Rv0565c activity within this entire lineage. Using the hypersensitivity phenotype in M. smegmatis, we tested the effect of the common R110H variant as well as a panel of *Rv0565c* amino acid variants found in ETH-resistant *M. tuberculosis* strains. Overexpression of Rv0565c containing the R110H variant conferred hypersensitization to ETH that was indistinguishable from the WT allele, suggesting that R110H does not alter Rv0565c function (Fig. 2b). In contrast, strains overexpressing allelic variant R87G, A272S, R296L, or V372D behaved like a strain carrying an empty vector, suggesting these mutations confer near-complete loss of function. Strains expressing allelic variant H95D, W172S, W254G, or N291H grew more robustly on low doses of ETH but not higher doses, suggesting these alleles confer partial loss of function (Fig. 2b; Fig. S3).

10.1128/mBio.00616-19.3 FIG S3 Growth of indicated M. smegmatis strains was monitored by spot plating of serial 10-fold dilutions of the indicated strains on solid medium containing various concentrations of ETH. Images were taken after 2.5 days of growth. Download FIG S3, PDF file, 10.5 MB. Copyright © 2019 Hicks et al. 2019 Hicks et al. This content is distributed under the terms of the Creative Commons Attribution 4.0 International license.

ETH activated by *ethA* is known to target *inhA* (16); however, it is possible that Rv0565c activation of ETH targets another essential process. To test whether Rv0565c-activated ETH similarly targets InhA, we constructed two additional strains overexpressing *inhA* either in a wild-type background or in an *ΔethA* strain with strong constitutive expression of *Rv0565c*, in which all ETH activation occurs through the activity of Rv0565c. As expected, *inhA* overexpression or *ethA* deletion alone increased growth on a restrictive concentration of ETH. In the sensitized *ΔethA*::*P_UV15_Rv0565c* strain, where all ETH activation is provided by Rv0565c, *inhA* expression also increased growth on ETH, consistent with InhA being the target of ETH activated by Rv0565c (Fig. 2c).

### Rv0565c contributes to ETH susceptibility in M. tuberculosis.

**FIG 3:**
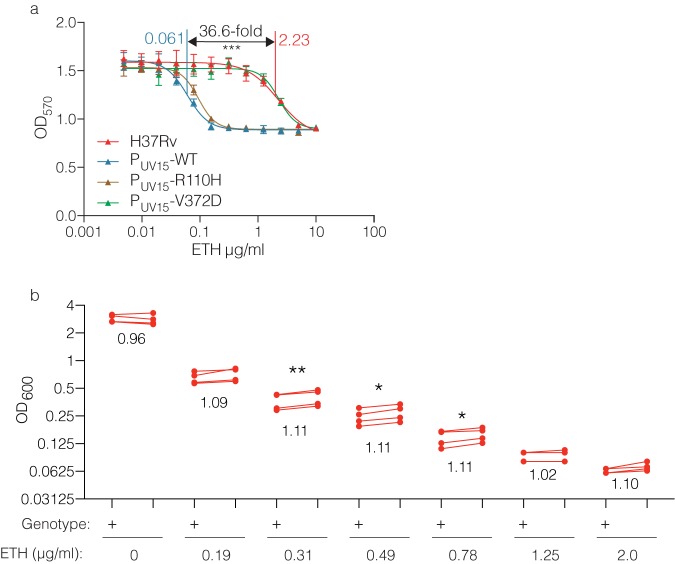
*Rv0565c* expression alters ETH susceptibility in H37Rv. (a) Growth of strains overexpressing *Rv0565c* wild-type or SNP-containing alleles in various concentrations of ETH as measured by alamarBlue reduction. Each point represents the mean and standard deviation from three technical replicates, and the line represents the nonlinear regression of growth dependent on concentration of antibiotic. (b) Comparison of the growth of H37Rv (+) and the H37Rv deletion of *Rv0565c* (Δ) at various concentrations of ETH. Each point represents the mean growth from three cultures within an experiment with each of four experimental replicates connected by a line. The number below each set of points is the average ratio of deletion over wild type across the 4 experiments. *, *P* < 0.05; **, *P* < 0.01, two-tailed paired *t* test comparing growth at each concentration.

Given the association between *Rv0565c* mutations and ETH resistance in clinical isolates as well as the ability of *Rv0565c* overexpression to confer susceptibility to ETH in M. smegmatis, we next assessed the effect of Rv0565c activity in *M. tuberculosis.* Similarly to M. smegmatis, overexpression of *Rv0565c* rendered the laboratory *M. tuberculosis* strain H37Rv hypersensitive to ETH, reducing the IC_50_ of ETH 36.6-fold as determined by alamarBlue reduction assay (Fig. 3a). Expression of the R110H allele conferred hypersensitivity similarly to expression of the wild-type allele, while the susceptibility of the strain expressing the V372D allele was indistinguishable from a strain carrying an empty expression vector.

Because the clinically prevalent mutations in Rv0565c appear to abrogate function, we next constructed an *Rv0565c* deletion strain in *M. tuberculosis* in the H37Rv background and compared its growth with wild-type H37Rv in ETH across a range of concentrations in which growth of the wild type is completely or partially inhibited. Concentrations which inhibited the growth of wild-type H37Rv were able to inhibit the growth of the *ΔRv0565c* strain. However, we observed that for a number of drug concentrations in which bacterial growth was partially inhibited (Fig. 3b), growth of the *ΔRv0565c* strain was modestly but significantly increased compared with the wild type across four biologically independent experiments performed on different days. As measured by area under the growth curve across concentrations (AUC), growth of the *ΔRv0565c* strain was on average 8.6% greater than the wild type (Fig. S4a and b) (range, 7.9% to 9.7%; two-tailed paired *t* test, *P* = 0.004).

10.1128/mBio.00616-19.4 FIG S4 (a and c) Representative graph of four biological replicates showing the growth of H37Rv and H37Rv *Rv0565c* deletion mutant (a) or 631 and 631 *Rv0565c* deletion mutant (c) in various concentrations of ETH. Points represent the mean from three technical replicates. (b and d) Paired area-under-the-curve measurements across 4 biological replicates for H37Rv (b) and strain 631 (d). The numbers to the right of each point represent the ratio of AUC of the deletion to wild type. **, *P* < 0.01, two-tailed paired *t* test of AUC values. Download FIG S4, PDF file, 0.8 MB. Copyright © 2019 Hicks et al. 2019 Hicks et al. This content is distributed under the terms of the Creative Commons Attribution 4.0 International license.

### Monooxygenase expression contributes to baseline ETH susceptibility in clinical isolates.

**FIG 4:**
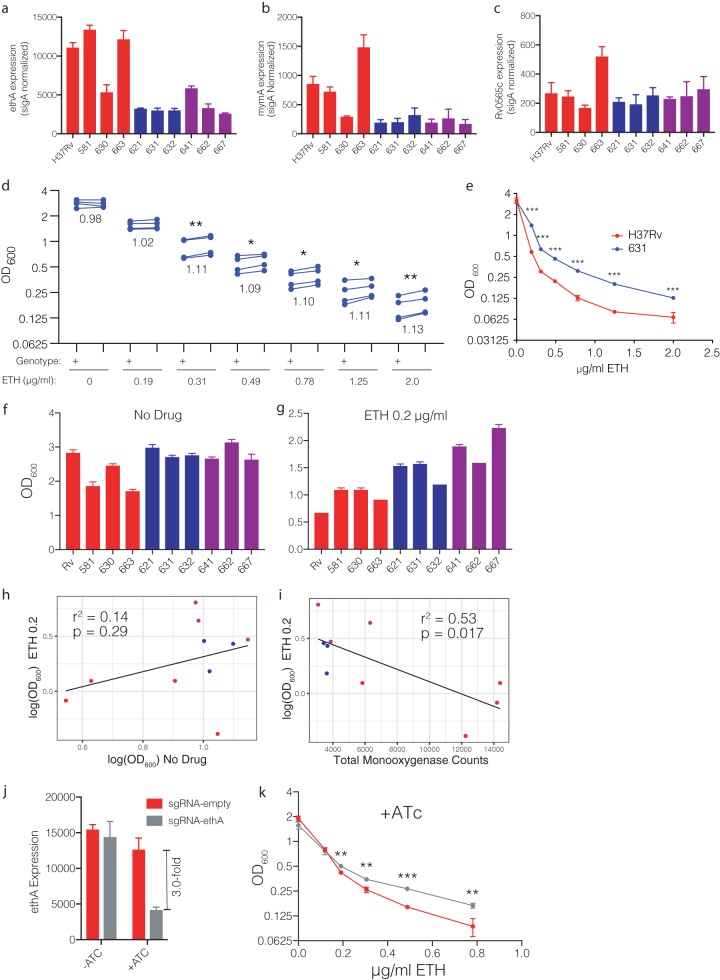
Monooxygenase expression affects baseline ETH susceptibility. (a to c) Expression of the monooxygenase genes *ethA*, *mymA*, and *Rv0565c* as measured by NanoString and normalized to expression of *sigA*. Each bar represents the mean normalized expression from three separate cultures, and error bars represent the standard deviation. Colors correspond to lineage designation, with red for lineage 4, blue for lineage 2, and purple for lineage 1. (d) Comparison of the growth of strain 631 (+) and the 631 deletion of *Rv0565c* (Δ) at various concentrations of ETH. Each point represents the mean growth from three cultures within an experiment with each of four experimental replicates connected by a line. The number below each set of points is the average ratio of deletion over wild type across the 4 experiments. *, *P* < 0.05; **, *P* < 0.01, two-tailed paired *t* test comparing growth at each concentration. (e) Growth of H37Rv and strain 631 across a range of ETH concentrations. Each point represents the mean OD_600_ from three cultures and is representative of 4 experiments. ***, *q* < 0.001, two-tailed *t* tests with false-discovery rate correction for multiple tests. (f and g) Growth of clinical isolates as measured by OD_600_ after 7 days of growth in either drug-free medium (f) or medium containing 0.2 μg/ml ETH (g). (h and i) Correlation of growth in 0.2 μg/ml ETH with growth in drug-free medium (h) or with the sum of monooxygenase expression across strains (i). The *r*^2^ and *P* values represent the fit of the linear regression plotted on each graph. (j) Knockdown efficiency of *ethA* in H37Rv as measured by NanoString. Each bar represents the mean *ethA* expression from three independent cultures normalized to *sigA* expression with error bars representing the standard deviation. (k) Growth of the H37Rv *ethA* knockdown strain compared with an empty guide control in the presence of ATc across a range of ETH concentrations. Each point represents the mean from three cultures. **, *q* < 0.01; ***, *q* < 0.001, two-tailed *t* tests with false-discovery rate correction for multiple tests.

Given the modest contribution of *Rv0565c* deletion to ETH susceptibility in H37Rv (lineage 4), we next considered whether the magnitude of the effect of *Rv0565c* deletion might depend on strain background. One GWA study of ETH resistance failed to correlate *Rv0565c* mutations with ETH resistance overall; however, the authors found that *Rv0565c* mutations were correlated with *ethA* mutations specifically in lineage 2/East Asian isolates (13). In our data set, the majority of mutations likewise occurred in lineage 2/East Asian strains; however, given the fact that this set of strains from China is dominated by lineage 2/East Asian strains, it was not possible to comment on whether this skewing was biologically meaningful. One possible explanation for differences in selective pressure among different strains would be if monooxygenases were expressed at different levels, leading to different relative contributions to ETH susceptibility compared with H37Rv. Using a panel of previously characterized clinical isolates (22) from lineages 1, 2, and 4, we examined expression of *ethA*, *mymA*, and *Rv0565c* (Fig. 4a to c). We observed that the expression levels of *ethA* and *mymA* were highly variable among clinical isolates, with all three lineage 2 isolates expressing <30% of the amount of each gene compared to H37Rv. Expression of *Rv0565c* was much less variable. However, given the lower expression of both *ethA* and *mymA* in lineage 2/East Asian isolates, we hypothesized that as a greater fraction of the monooxygenase pool, Rv0565c might make a greater contribution to ETH susceptibility in lineage 2/East Asian strains. To test this hypothesis, we constructed an *Rv0565c* deletion mutant in the lineage 2 clinical isolate 631. Deletion of *Rv0565c* had a remarkably similar effect on ETH susceptibility in 631 as in H37Rv where growth was increased by ∼10% across a range of partially inhibitory concentrations, albeit across a wider range of concentrations (Fig. 4d). The AUC in partially inhibitory concentrations of ETH was increased on average by 11.5% (Fig. S4c and d) (range, 7.9% to 16.3%; two-tailed paired *t* test, *P* < 0.01). Thus, *Rv0565c* deletion conferred increased growth in partially inhibitory concentrations of ETH in both H37Rv and East Asian strain 631, but these data did not suggest a larger role for Rv0565c in lineage 2 isolates.

In these analyses, we noted that there was a substantial difference in the growth of wild-type H37Rv and *M. tuberculosis* strain 631 in the same ETH concentrations (Fig. 4e). Given the previously proposed model that EthA and MymA make independent contributions to ETH activation, we hypothesized that growth in partially inhibitory concentrations of ETH may be explained by the total expression of EthA, MymA, and Rv0565c among clinical isolates. Growth among strains was variable in the absence of antibiotics, as well as in the presence of 0.2 μg/ml ETH (Fig. 4f and g). However, growth in the presence of ETH was not statistically correlated with growth in no-drug medium (Fig. 4h) (*r*^2^ = 0.14, *P* = 0.29). In contrast, total monooxygenase expression was negatively correlated with growth in the presence of ETH (Fig. 4i) (*r*^2^ = 0.53, *P* = 0.017). Monooxygenase expression remained negatively correlated with growth in the presence of ETH even when controlling for growth in no-drug medium using multiple regression (no-drug growth, *P* = 0.24; monooxygenase expression, *P* = 0.020). This correlation was also observed at higher doses of ETH in which growth was further suppressed (Fig. S5). To further test the model that differences in the total level of monooxygenase expression affect *M. tuberculosis* growth in partially inhibitory concentrations of ETH, we conditionally knocked down *ethA* expression in H37Rv using CRISPR-mediated interference (23). Consistent with the model where baseline monooxygenase expression affects growth at low doses of ETH, modest (∼3-fold) *ethA* depletion significantly increased growth in partially inhibitory concentrations of ETH (Fig. 4j and k; Fig. S6). Importantly, our strains have no variants within *ethA*, its promoter, the *ethA* regulator *ethR*, or the putative promoter regions upstream of *mymA* and *Rv0565c* (22), which could explain the differences in monooxygenase expression levels (24). This finding suggests that there are as-yet-unrecognized genetic variants that underlie the differences in total monooxygenase expression and modulate ETH susceptibility.

10.1128/mBio.00616-19.5 FIG S5 (a) Growth of clinical isolates as measured by OD_600_ after 7 days of growth in medium containing 1.8 μg/ml ETH. (b) Correlation of growth in 1.8 μg/ml ETH with the sum of monooxygenase expression across strains. The *r*^2^ and *P* values represent the fit of the linear regression. Download FIG S5, PDF file, 0.4 MB. Copyright © 2019 Hicks et al. 2019 Hicks et al. This content is distributed under the terms of the Creative Commons Attribution 4.0 International license.

10.1128/mBio.00616-19.6 FIG S6 Growth of the H37Rv *ethA* knockdown strain compared with an empty guide control in the absence of ATc induction across a range of ETH concentrations. Two-tailed *t* tests with false-discovery rate correction for multiple tests are annotated above each concentration. Download FIG S6, PDF file, 0.4 MB. Copyright © 2019 Hicks et al. 2019 Hicks et al. This content is distributed under the terms of the Creative Commons Attribution 4.0 International license.

### Rv0565c expression makes a significant contribution to prothionamide susceptibility.

We next considered other explanations for the reported association of *Rv0565c* mutations specifically with lineage 2/East Asian *M. tuberculosis* strains in a global data set. In China, the ethionamide analog prothionamide (PTH) is used in place of ETH for second-line treatment because PTH is manufactured in China (Y. Zhao, personal communication). These antibiotics are considered interchangeable in MDR regimen design (14); however, studies directly comparing isolate susceptibility to ETH with that to PTH consistently show a lower MIC for PTH (25, 26), suggesting that they are not completely biologically equivalent. To test whether Rv0565c may contribute more to PTH susceptibility than to ETH susceptibility, we repeated the characterization of *M. tuberculosis Rv0565c* mutants during exposure to PTH.

**FIG 5:**
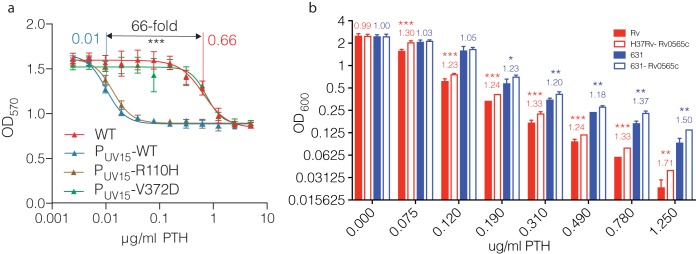
Rv0565c expression more substantially alters PTH susceptibility. (a) Growth of strains overexpressing *Rv0565c* wild-type or SNP-containing alleles in various concentrations of PTH as measured by alamarBlue reduction. Each point represents the mean and standard deviation from three technical replicates, and the line represents the nonlinear regression of growth dependent on concentration of antibiotic. (b) Growth of H37Rv and strain 631 with their corresponding *Rv0565c* deletion mutants in various concentrations of PTH. Bar height represents the mean from three independent cultures with error bars showing standard deviations. The number above the bars is the ratios of the average of mutant over wild-type OD_600_. Data are representative of two experiments. *, *q* < 0.05; **, *q* < 0.01; ***, *q* < 0.001; two-tailed *t* tests with false-discovery rate correction for multiple tests.

Consistent with the observed difference in ETH and PTH activity in previous studies of clinical isolates, the IC_50_ of PTH was 0.66 μg/ml compared with 2.23 μg/ml for ETH in H37Rv. Unexpectedly, overexpression of *Rv0565c* in H37Rv reduced the IC_50_ of PTH to 0.01 μg/ml, a change of 66-fold, which was 1.8-fold greater than the 36.6-fold change observed for ETH comparing the same strains (Fig. 5a). Deletion of *Rv0565c* in H37Rv likewise conferred a larger growth advantage in PTH than in ETH (Fig. 5b), where the deletion grew 23 to 71% more across partially inhibitory concentrations of PTH (Fig. 5b) and the AUC for the *Rv0565c* deletion mutant was on average 26.9% greater in partially inhibitory concentrations of PTH, which was significantly greater than the 8.6% increase in ETH (Tukey’s multiple-comparison test, adjusted *P* = 0.0003). This finding was replicated in East Asian strain 631, where deletion of *Rv0565c* increased growth by 18 to 50% in partially inhibitory concentrations (Fig. 5b) and increased the AUC in PTH by 20.7% compared with 11.5% in ETH (Tukey’s multiple-comparison test, adjusted *P* = 0.02). Taken together, these data suggest that Rv0565c activity has a greater effect on PTH susceptibility than on ETH susceptibility, perhaps due to enzyme affinity, and programmatic differences in the use of ETH versus PTH may explain geographic differences in the emergence of resistance mutations.

**FIG 6:**
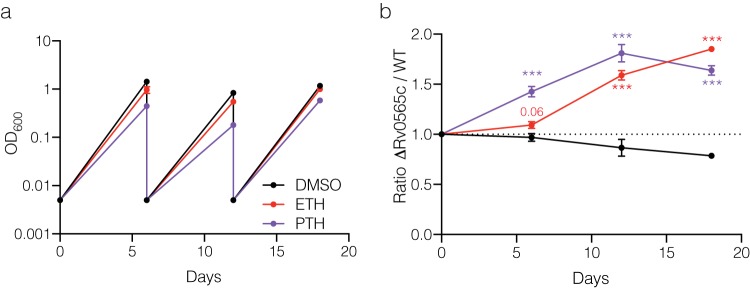
Rv0565c deletion in strain 631 confers a long-term growth benefit in ETH and PTH. (a) Growth of a pooled mixture of strain 631 and 631 Rv0565c deletion mutant in the indicated medium as measured by OD_600_. Strains were rediluted to 0.005 every 6 days. (b) The ratio of Rv0565c mutant over wild-type strain 631 over the course of treatment normalized to the starting library ratio on day 0. Each point represents the mean value from three qPCR technical replicates of three independent cultures with error bars showing the standard deviations among the three cultures. Each time point was compared with the input ratio by multiple *t* tests after two-way ANOVA and corrected for multiple hypothesis testing. ***, *q* < 0.001.

To further corroborate our finding that deletion of Rv0565c confers a meaningful growth advantage in the presence of antibiotic pressure, we performed long-term competition assays using strain 631 and the 631 *Rv0565c* deletion strain. Strains were cocultured in the absence of antibiotic selection or in the presence of ETH or PTH at 0.25 μg/ml, which partially inhibited growth (Fig. 6a). Using qPCR of genomic DNA targeting regions specific to each strain to quantify the relative abundance, we observed that by 6 days of exposure, the deletion mutant became 10% more abundant in ETH while in PTH it became 40% more abundant (Fig. 6b). This measurement was remarkably consistent with that taken by OD_600_ readings for individual strains (Fig. 4d and Fig. 5b). Upon redilution and continued growth, the differences in abundance expanded to 59% and 81% in ETH and PTH, respectively, by 12 days, and by 18 days the deletion mutant was 85% and 64% more abundant, respectively (Fig. 6b). These findings demonstrate that the relatively small advantage conferred by *Rv0565c* deletion can be expanded over longer antibiotic exposures.

## DISCUSSION

The intense selective pressure of antibiotic therapy against TB has led to the evolution of strains with a diverse set of mutations undermining treatment. Although the gold standard for measuring antibiotic susceptibility is growth-based phenotypic testing, genotype-based diagnostics are increasingly offering a more rapid and sensitive approach in cases where the full spectra of mutations underlying resistance are understood. For the widely used second-line antibiotic ethionamide (ETH), however, mutations in *ethA* and *inhA* and its promoter are able to predict phenotypic resistance with only ∼65% sensitivity (5). The recent discovery of *ethA* promoter mutants (27) and the implication of *mymA* in ETH susceptibility (20) promise to increase this sensitivity; however, it remains unclear whether this list is exhaustive. Here we examine the genomes of clinical isolates phenotyped for ETH resistance and identify several loci associated with resistance, including the monooxygenase *Rv0565c*. By overexpression of *Rv0565c*, we clearly demonstrate that it is an activator of ETH and PTH in *M. tuberculosis*, and mutations observed in clinical isolates confer partial or complete loss of function. Nevertheless, deletion of *Rv0565c* confers only a small growth advantage during treatment with ETH or PTH *in vitro*. This leads us to consider three alternate hypotheses regarding the impact of these mutations on antibiotic efficacy *in vivo*.

First, it is possible that our *in vitro* conditions poorly capture the true effect of Rv0565c activity *in vivo* and thus the effect of *Rv0565c* mutations. Expression of *Rv0565c* is low compared with *ethA* and *mymA* during growth in standard 7H9 medium, based on NanoString (Fig. 4) and qPCR analysis (data not shown). Thus, deletion of *Rv0565c* likewise can confer only a modest benefit *in vitro*. However, under any condition in which expression of *Rv0565c* is upregulated, wild-type strains may more closely resemble the *Rv0565c* overexpression strains, where introduction of a loss-of-function polymorphism may then dramatically increase resistance to ETH/PTH. Indeed, *Rv0565c* appears to be upregulated in macrophages while *mymA* is downregulated (28), suggesting that characterization of various monooxygenase mutants in infection models may help elucidate their relative roles in different contexts.

Second, although deletion of *Rv0565c* in the lineage 2 clinical isolate 631 failed to confer a more substantial shift in ETH IC_50_ compared with the laboratory strain H37Rv, it is possible that the balance of monooxygenase expression in other clinical isolates, even as measured *in vitro*, favors mutations in *Rv0565c* or *mymA* rather than the canonical activator *ethA.* Supporting this hypothesis, one study of serial isolates derived from individual patients during treatment identified acquisition of an *Rv0565c* mutation coinciding with the conversion from ETH susceptible to ETH resistant by standard phenotypic susceptibility testing (29).

Finally, it is possible that small changes in the ability of strains to grow in the presence of ETH or PTH may confer enough of an advantage to be selected for over 18 to 24 months of second-line antibiotic therapy. This selective advantage may not be trivial from the perspective of patient and caregiver, as Colangeli et al. recently demonstrated that small differences in isoniazid and rifampin MICs among supposedly susceptible strains could partially explain treatment outcome in a small cohort of patients (10). In this case, the differences in growth in partially inhibitory concentrations of ETH and PTH among clinical isolates based upon the expression of their monooxygenases may also prove an important determinant of treatment outcome. Examination of the *ethA* promoter and the known regulator *ethR* revealed no variation among the panel of clinical isolates which we studied; however, the underlying genetic determinants of *ethA* expression variation could now be identified using a genome-wide association approach directly correlating genetic variants with *ethA* expression.

In any of these three models, the integration of GWA and experimental data suggest the importance of incorporating *Rv0565c* mutations into decisions about the use of ethionamide and prothionamide in MDR treatment regimens. Although it is clear that high-level resistance as measured *in vitro* is a partial predictor of treatment response, small changes in MIC conferred by *Rv0565c* mutation and activator expression, as well as condition-dependent phenotypes experimentally defined in the context of genome-wide associations in clinical isolates, may allow us to refine treatment choices.

## MATERIALS AND METHODS

### Genomic data analysis and genetic association.

Genomic data were downloaded from the NCBI Sequence Read Archive accession numbers SRA065095 and PRJNA268900. To identify genetic variants, paired-end data were aligned against the H37Rv reference genome NC_00962.3 using the bwa mem algorithm implemented in bwa 0.9.7a (30), and variants were identified using the Genome Analysis Toolkit (31) version 3.5 haplotype caller set for haploid genomes and emitting reference confidence calls at base-pair resolution. Base-pair-resolution genomic variant call formats (gVCFs) were merged to make a master VCF from which variants were identified using the GenotypeGVCFs tool. For phylogenetic and genetic association analysis, the variants were filtered to retain only SNPs, which were annotated using snpEff (32). For phylogenetic analysis, the Mycobacterium canettii strain CIPT 140010059 genome (NC_15848.1) was used as an outgroup. To identify variants separating *M. canettii* from H37Rv, the wgsim function of SAMtools (33) was used to simulate 3 million 72-bp-paired end reads which were then analyzed like any other sample.

To construct the phylogenetic tree, SNPs in repetitive regions of the genome or regions known to confer antibiotic resistance were removed as previously described (9). To remove low-confidence calls, a manual threshold was set to call either a variant or reference alleles, where any site in any sample not achieving 10× read coverage with at least 80% of bases in agreement was masked as missing data. In addition, any site in which fewer than 90% of isolates achieved a high-confidence call was removed from the analysis. Phylogenetic tree reconstruction was performed using RAxML (34) v8.2.11 with the GTRCAT model of nucleotide evolution and rooted using *M. canettii* as the outgroup. Trees were visualized using the online interactive Tree of Life server (35). Ancestral sequence reconstruction was performed with RAxML v8.2.11 as well to determine the most probable ancestral allele at each SNP site. To determine the number of times each mutation evolved in the data set, the alleles were mapped back onto the whole-genome phylogenetic tree and the parsimony score was calculated with the Fitch algorithm as implemented in the R package Phangorn (36).

Genome-wide associations were calculated as previously described using the phyOverlap algorithm (9). Sites which differed from H37Rv only in *M. canettii* were excluded from analysis, as were synonymous mutations. Enrichment scores for each gene are calculated as the fraction of isolates containing a variant that are also resistant to ETH averaged across all sites within a gene weighted by the number of times a mutation evolves across the phylogenetic tree. This value is a standard output of phyOverlap. False-discovery rate correction was performed using the Benjamini-Hochberg procedure. Strains without a phenotypic drug susceptibility test (DST) for ethionamide (*n* = 15) were removed from this analysis.

Further analysis of SNPs and indels affecting *Rv0565c*, *mymA*, and *ethA* shown in Fig. 1 and in Table S1a to c in the supplemental material was performed by manual inspection of the variant matrix produced by Genome Analysis Toolkit (GATK) which includes both types of variants.

To examine *ethA*, *ethR*, and their promoters in the panel of clinical isolates, the genomes assembled in the work of Carey et al. (22) were downloaded from the SRA database. Alignment was performed for each gene, including 100 bp upstream of the open reading frame, against all genomes using the online BLAST tool megablast with default settings (37).

### Bacterial growth.

Mycobacterium smegmatis strains were grown and maintained in 7H9 Middlebrook medium supplemented with 0.2% glycerol, 5 g/liter BSA fraction V, 2 g/liter dextrose, 0.85 g/liter sodium chloride, and 0.05% Tween 80. Plating assays were performed on 7H10 Middlebrook medium supplemented with 0.5% glycerol, 5 g/liter BSA fraction V, 2 g/liter dextrose, 0.85 g/liter sodium chloride, and 0.05% Tween 80 and antibiotics at the indicated concentrations.

Mycobacterium tuberculosis was grown and maintained in 7H9 Middlebrook medium supplemented with 0.2% glycerol, 10% Middlebrook oleic acid-albumin-dextrose-catalase (OADC), and 0.05% Tween 20.

### Strain and mutant construction.

M. smegmatis strains were constructed in the mc^2^155 strain background. Deletion of *Msmeg_2038* and *ethA^Msmeg^* was performed using recombineering as described by Murphy et al. (38). Briefly, a hygromycin resistance cassette flanked with *lox* sites and then 500 bp of sequence flanking the start and stop codon of each gene was electroporated into mc^2^155 expressing the RecET phage proteins. Transformants were selected on hygromycin-containing medium and PCR validated for replacement of the gene of interest with the resistance cassette. For the *Msmeg_2038* deletion, the hygromycin cassette was subsequently removed by expression of Cre recombinase. Overexpression strains of *rv0565c* and *inhA* were constructed by integration of a kanamycin-resistant plasmid at the L5 phage integration site containing the gene of interest under the control of the constitutive UV15 promoter.

H37Rv and strain 631 mutants with deletions of *rv0565c* were constructed using the ORBIT method (39). Strains carrying the *recT* expression vector pKM461 were cotransformed with the ORBIT oligonucleotide GATCTGGAGGGGCTGGCTGGATGTCCTGGCTACCCTGGTCGCTGATCCAGGATTTCAAGCGAGGTTCACG[GGTTTGTCTGGTCAACCACCGCGGTCTCAGTGGTGTACGGTACAAACC]TTGCTCCTGCGGTTCGTCGACGGATATCAAATGTCCGGTTGCCGCAATACGCTGAACGCCATGGCAGCCA (with the *attB* site bracketed) and pKM464, which results in a total deletion of *rv0565c* and replacement with the pKM464 hygromycin-resistant plasmid. Overexpression M. tuberculosis strains of *Rv0565c* and SNP variants were constructed by integration of a kanamycin-resistant plasmid at the L5 phage integration site containing the gene of interest under the control of the constitutive UV15 promoter.

CRISPR-mediated knockdown was performed as described by Rock et al. (23) using the pJR965 vector. The *ethA* knockdown strain was transformed with the guide targeting GGCGGCCGTGTACAGCGCCTG while the control strain was transformed with the unmodified pJR965.

### Antibiotic susceptibility testing.

Antibiotic susceptibility testing was performed for M. smegmatis by growing the indicated strains to mid-logarithmic phase, diluting each to an OD_600_ of 0.2, and then spotting 2 μl (Fig. 2), 5 μl (Fig. S2), or 8 μl (Fig. S3) of serial 10-fold dilutions from undiluted to 10^−4^ on 7H10 agar as described above with antibiotics at the indicated concentrations. Images were taken after 2 to 3 days of growth as indicated in the figure legends.

Antibiotic susceptibility testing of M. tuberculosis strains overexpressing *rv0565c* was performed using the alamarBlue reduction to measure growth. Indicated strains were grown to mid-logarithmic phase and then seeded at an OD_600_ of 0.0005 in 200 μl of 7H9 OADC medium with antibiotics at the indicated concentration. After 4 days of growth with shaking at 37°C, 20 μl of alamarBlue (Bio-Rad) was added and the plates were incubated for an additional day. Reduction of alamarBlue was measured by absorbance at OD_570_.

Growth of *Rv0565c* deletion strains in antibiotics was performed in a similar manner. Indicated strains were grown to mid-logarithmic phase and then diluted to 0.01 in 600 μl of medium containing ETH or PTH at serial 1.6-fold dilutions. The concentrations of ETH (Fig. 3b) ranged from 2 to 0.12 μg/ml. For PTH (Fig. 5b), a lower range was used due to the increased potency of PTH, with the concentrations ranging from 1.25 to 0.075 μg/ml. Optical density was measured after 6 days of growth with shaking at 37°C. For experiments with CRISPR-mediated knockdown, anhydrous tetracycline (ATc) was added at a final concentration of 100 ng/ml to strains 2 days prior to dilution into antibiotic medium and was maintained throughout the assay. For clinical isolates, the protocol was slightly modified by reducing the input dilution to 0.005 and strains were allowed to grow for 7 days in drug-containing medium before final optical density was read.

### Gene expression analysis.

RNA was extracted as previously described (22). Briefly, strains were grown to mid-logarithmic phase, spun down at 4,000 rpm for 10 min, and resuspended in TRIzol reagent. Cells were disrupted by bead beating, and 1/5 volume of chloroform was added. RNA was extracted from the TRIzol lysate using the Direct-zol miniprep kit (Zymo Research), with an additional in-tube DNA digestion with Turbo DNase (ThermoFisher Scientific) to remove residual DNA. Expression was assessed using the nCounter chemistry on the NanoString Sprint platform with 25 ng of total RNA as input. Expression of monooxygenases was normalized to expression of *sigA* within each sample using the nSolver software (NanoString Technologies). Hybridization probes used for transcript counting are included in Table S3.

10.1128/mBio.00616-19.9 TABLE S3 NanoString probes for the detection of *sigA*, *Rv0565c*, *mymA*, and *ethA* (Fig. 4a to c). Download Table S3, XLSX file, 0.01 MB. Copyright © 2019 Hicks et al. 2019 Hicks et al. This content is distributed under the terms of the Creative Commons Attribution 4.0 International license.

### Statistical analysis.

alamarBlue reduction curves (Fig. 3a and Fig. 5a) were analyzed in Prism 7 (GraphPad Software). Antibiotic concentrations were log transformed, and then nonlinear regression was performed using a 4-parameter curve to fit the OD_570_ values and estimate the IC_50_ for each strain with three technical replicates for each strain and drug concentration. Growth of strains at partially inhibitory concentrations of ETH and PTH in Fig. 3b and Fig. 4d was performed using three independent cultures across 4 separate experiments. Testing for differences in growth was assessed by two-tailed paired *t* tests with data from each of the 4 experiments paired to account for day-to-day variation in overall growth. Differences in growth shown in Fig. 4e and Fig. 5b were tested by multiple *t* tests comparing 3 cultures within a single experiment using the Benjamini, Krieger, and Yekutieli method as implemented by Prism 7. These measurements are representative of 4 and 2 experiments, respectively. Area-under-the curve (AUC) measurements shown in Fig. S4 were assessed for each biological replicate consisting of three technical replicates with separate wells for each strain. No-drug measurements were excluded from this analysis to measure the effect of genotype on growth in the presence of partially inhibitory concentrations of antibiotic. Comparison of growth among genotypes across biological replicates was performed with a two-sided paired *t* test of the AUC as implemented in Prism 7. Comparison of ETH and PTH AUC ratios was performed by one-way ANOVA followed by Tukey’s multiple-comparison test. For both H37Rv and 631, the 4 biological replicates comparing each with its corresponding deletion in ETH were contrasted with the 2 biological replicates in PTH.

### Competition growth assays.

Strain 631 and the 631 Rv0565c deletion were mixed 1:1 after expansion in antibiotic-free medium. The pooled mixture was then subcultured to an OD_600_ of 0.005 in 25 ml of medium containing ETH at 0.25 μg/ml, PTH at 0.25 μg/ml, or DMSO at an equal volume. Every 6 days for 18 days, the OD was measured and strains were rediluted to 0.005 in 25 ml of the corresponding medium. Simultaneously, genomic DNA (gDNA) was extracted from 10 ml of culture using a phenol-chloroform extraction protocol. Briefly, cultures were spun down at 4,000 rpm at room temperature. The supernatant was discarded, and pellets were resuspended in 700 μl TE-NaCl (10 mM Tris-HCl, pH 8.0, 1 mM EDTA, 50 mM NaCl) and transferred into bead-beating tubes. Three hundred microliters of 50:50 phenol-chloroform, pH 8, was then added, and cells were disrupted by two rounds of beating on a MagNA Lyser at setting 6.5 for 30 s each. Phase separation was performed by spinning in a microcentrifuge for 10 min at 4°C. The upper aqueous layer was transferred to a new tube to which RNase A was added at a final concentration of 80 μg/ml and allowed to incubate at 37°C for 30 min. Three hundred microliters of phenol-chloroform was then added again, and samples were removed from the BSL-3 laboratory. After another phase separation, the aqueous supernatant was mixed with 1/10 volume of 3 M sodium acetate, pH 5.2, and 1 volume of isopropanol. Samples were incubated at −20°C for >3 h and then precipitated by centrifugation at 14,000 rpm at 4°C for 15 min. DNA pellets were washed with 1 ml of 70% ethanol and resuspended in 10 mM Tris HCl, pH 8. DNA was quantified by Qubit assay, and then the relative abundance of each strain was measured by two qPCRs targeting the gene *Rv0565c* and the hygromycin resistance cassette, which are specific to the wild-type and Rv0565c deletion strains, respectively.

The abundance of each gene was measured for three independent cultures at each time point in technical qPCR triplicates using 0.45 ng of DNA as input. The relative abundance of each strain was calculated using the ΔΔ*C_T_* method. First, the average *C_T_* of hygromycin was subtracted by the average Rv0565c *C_T_* for each culture, and then the average difference in the input library was subtracted from this value to account for differences at time zero. The fold abundance of mutant over wild type was then calculated as 2^ΔΔ^*^CT^* in each culture. Each time point was compared with the input ratio by multiple two-tailed *t* tests after two-way ANOVA and corrected for multiple hypothesis testing using Prism 8. qPCR was performed on the Applied Biosystems Viia 7 platform using the iTaq Sybr Green supermix (Bio-Rad). The reaction mixtures were initially denatured for 3 min at 95°C, and then 40 cycles of PCR were performed with 30 s at 95°C and 45 s at 62°C followed by a melt curve. All samples exhibited unimodal melting profiles consistent with specific amplification. The Rv0565c detection primers were 5′-CAACTATGACGAGGGCTACAC-3′ and 5′-CCGATCACCACGATCTTCTT-3′. The hygromycin cassette detection primers were 5′-GTGACACAAGAATCCCTGTTACT-3′ and 5′-CCAGAATTCCTGGTCGTTCC-3′.
