# Supplementary material for: Bacterial Genome-Wide Association Identifies Novel Factors That Contribute to Ethionamide and Prothionamide Susceptibility in Mycobacterium tuberculosis
Source: mBio. 2019 Apr 23;10(2):e00616-19. doi: 10.1128/mBio.00616-19 (PMC6479004; doi:10.1128/mBio.00616-19)
Supplement: FIG S1 [file mBio.00616-19-sf001.pdf]

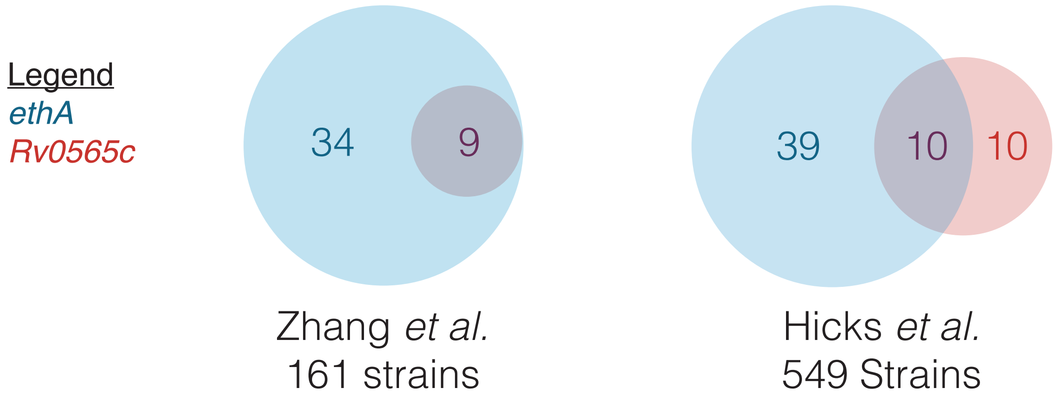

Supplementary Figure 1.
The overlap of Rv0565c and ethA mutations among strains from Zhang *et al*. and Hicks *et al (2018)*. The common, lineage associated mutations *Rv0565c*-R110H, *ethA*-S266R, and *ethA*-P334A were excluded from this analysis.
