# Supplementary material for: Bacterial Genome-Wide Association Identifies Novel Factors That Contribute to Ethionamide and Prothionamide Susceptibility in Mycobacterium tuberculosis
Source: mBio. 2019 Apr 23;10(2):e00616-19. doi: 10.1128/mBio.00616-19 (PMC6479004; doi:10.1128/mBio.00616-19)
Supplement: FIG S3 [file mBio.00616-19-sf003.pdf]

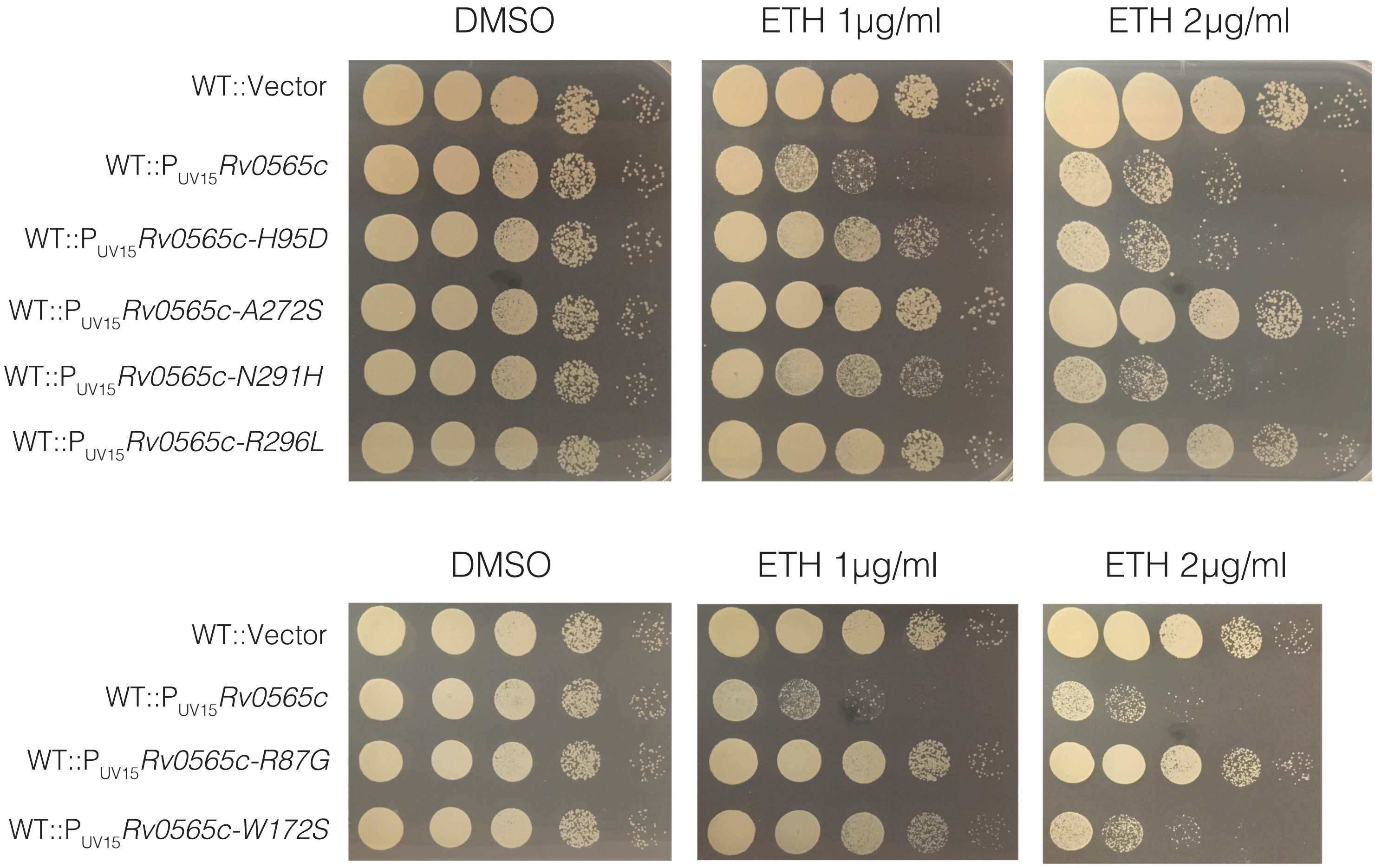

Supplementary Figure 3
Growth of indicated M. smegmatis strains was monitored by spot plating of serial 10-fold dilutions of the indicated strains on solid media containing varying concentrations of ETH. Images were taken after 2.5 days of growth.
