# Supplementary material for: Bacterial Genome-Wide Association Identifies Novel Factors That Contribute to Ethionamide and Prothionamide Susceptibility in Mycobacterium tuberculosis
Source: mBio. 2019 Apr 23;10(2):e00616-19. doi: 10.1128/mBio.00616-19 (PMC6479004; doi:10.1128/mBio.00616-19)
Supplement: FIG S4 [file mBio.00616-19-sf004.pdf]

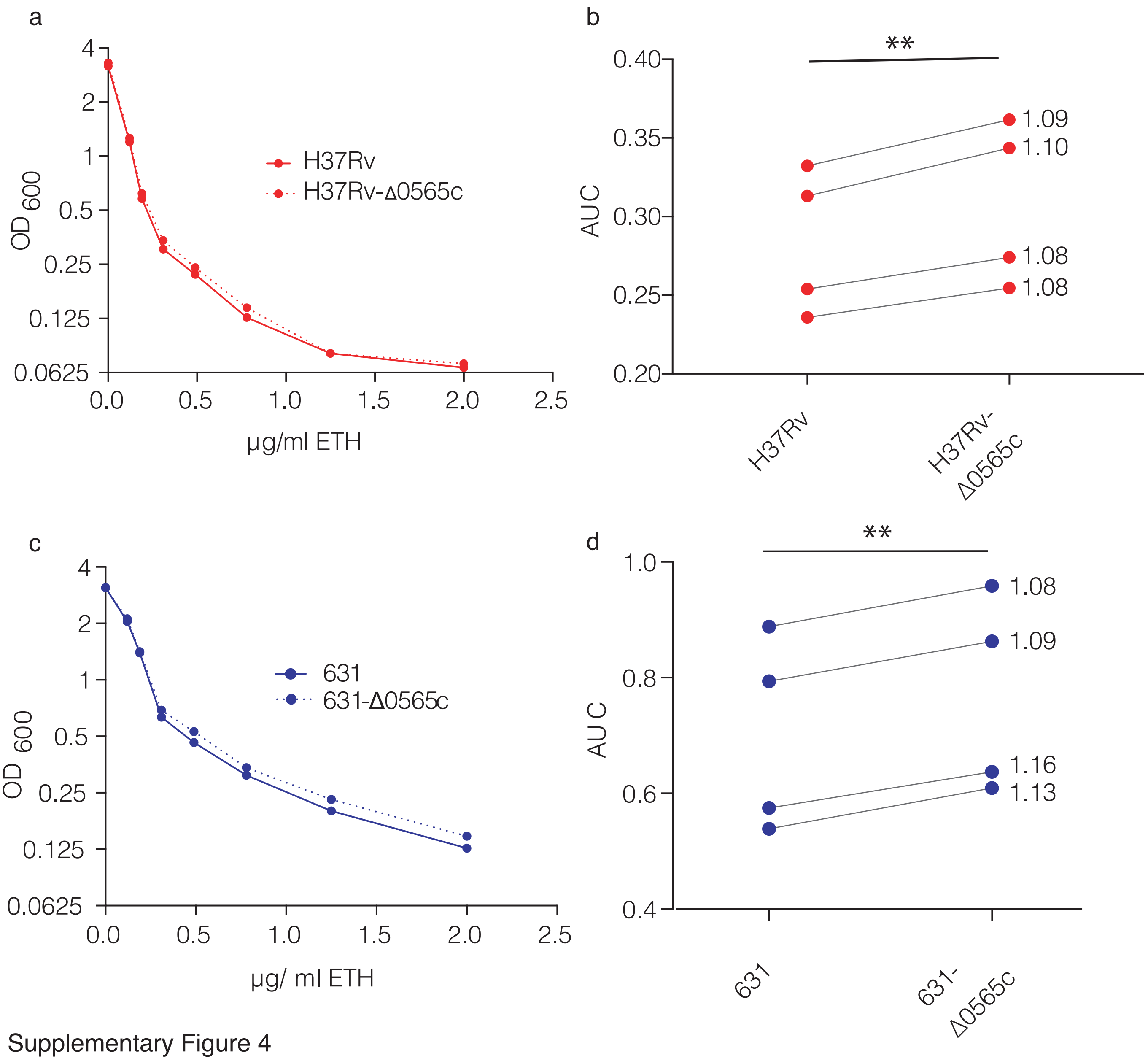

Supplementary Figure 4
(a,c) Representative graph of four biological replicates showing the growth of H37Rv and H37Rv Rv0565c deletion mutant (a) or 631 and 631 Rv0565c deletion mutant (c) in varying concentrations of ETH . Points represent the mean of three technical replicates. (b,d) Paired area-under-the-curve measurements across 4 biological replicates for H37Rv (b) and strain 631 (d). The numbers to the right of each point represent the ratio of AUC of the deletion to wildtype. ** $p < 0.01$ two-tailed paired t-test of AUC values.
