# Supplementary material for: Bacterial Genome-Wide Association Identifies Novel Factors That Contribute to Ethionamide and Prothionamide Susceptibility in Mycobacterium tuberculosis
Source: mBio. 2019 Apr 23;10(2):e00616-19. doi: 10.1128/mBio.00616-19 (PMC6479004; doi:10.1128/mBio.00616-19)
Supplement: FIG S5 [file mBio.00616-19-sf005.pdf]

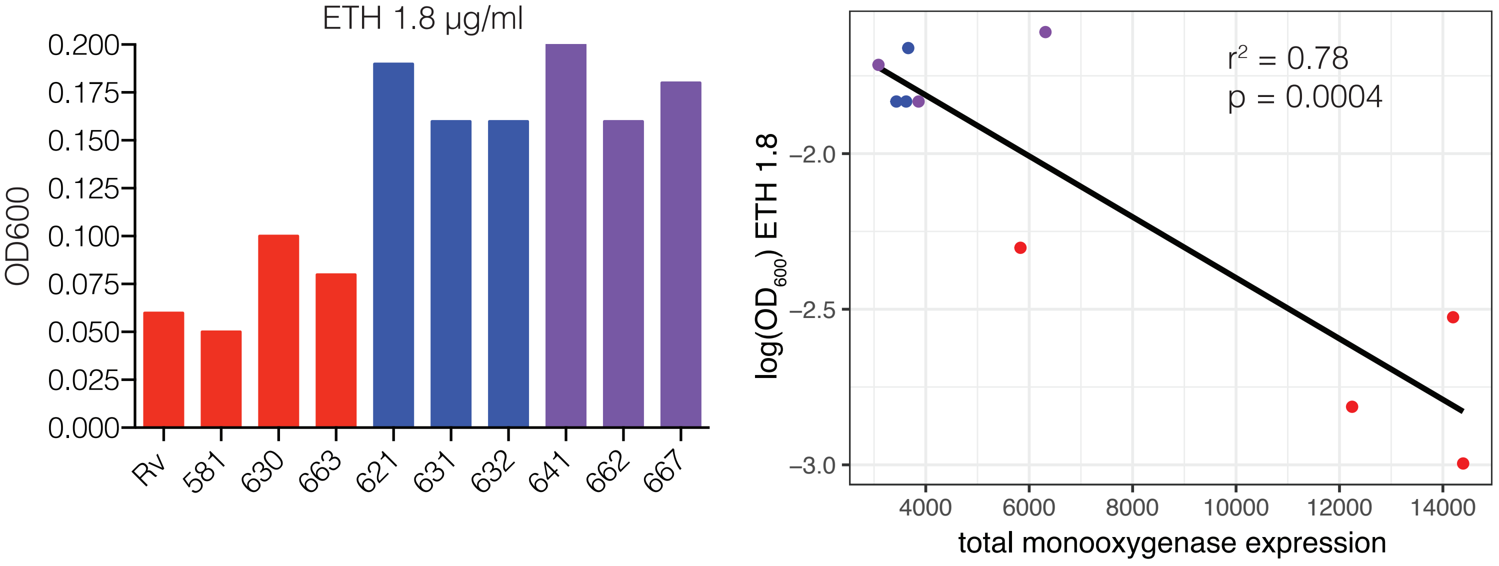

Supplementary Figure 5

(a) Growth of clinical isolates as measured by OD600 after 7 days of growth in media containing 1.8 μg/ml ETH. (b) Correlation of growth in 1.8 μg/ml ETH with the sum of monooxygenase expression across strains. The r^2 and p-values represent the fit of the linear regression.
