# Supplementary material for: Bacterial Genome-Wide Association Identifies Novel Factors That Contribute to Ethionamide and Prothionamide Susceptibility in Mycobacterium tuberculosis
Source: mBio. 2019 Apr 23;10(2):e00616-19. doi: 10.1128/mBio.00616-19 (PMC6479004; doi:10.1128/mBio.00616-19)
Supplement: FIG S6 [file mBio.00616-19-sf006.pdf]

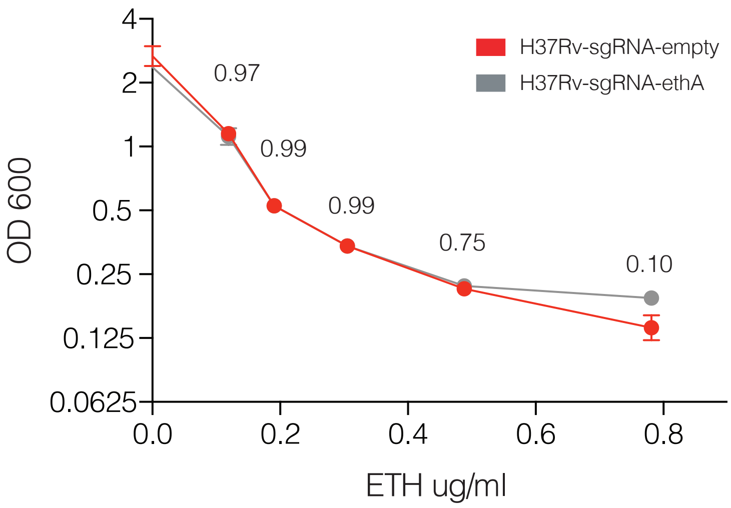

Supplementary Figure 6
Growth of the H37Rv ethA knockdown strain compared with an empty guide control in the absence of ATc induction across a range of ETH concentrations. Two-tailed t-tests with false-discovery rate correction for multiple tests are annotated above each concentration.
